# Factor analysis of the adolescent version of the Eating Disorders Examination Questionnaire (EDE-Q): results from Swedish general population and clinical samples

**DOI:** 10.1186/s40337-017-0140-8

**Published:** 2017-06-01

**Authors:** Emma Forsén Mantilla, Andreas Birgegård, David Clinton

**Affiliations:** 1 Department of Clinical Neuroscience, Karolinska Institutet, and Centre for Psychiatry Research, Stockholm County Council, Norra stationsgatan 69, 7 tr, 113 64 Stockholm, Sweden; 2 Institute for Eating Disorders, Kruses gata 8, 0263 Oslo, Norway

**Keywords:** Eating disorders, Factor analysis, Eating Disorder Examination Questionnaire, Assessment, Measurement, Adolescent

## Abstract

**Background:**

Although the Eating Disorder Examination Questionnaire (EDE-Q) is perhaps the single most widely used self-report measure of eating disorder (ED) symptoms, important questions remain about its validity and applicability in relation to particular groups of individuals, especially adolescents of both genders.

**Methods:**

The present study investigated the factor structure of the adolescent version of the Eating Disorder Examination Questionnaire (EDE-Q) in samples of Swedish girls and boys from the general population and girls with a diagnosed eating disorder. Girls (*N* = 239) and boys (*N* = 248) aged between 13 and 15 years who were attending school, and girls (*N* = 570) aged between 12 and 14 years who were in treatment for an eating disorder at a specialist eating disorder clinic were assessed on the adolescent version of the EDE-Q.

**Results:**

The adolescent version of the EDE-Q demonstrated satisfactory levels of internal consistency. However, confirmatory factor analysis (CFA) failed to support the four-factor model of the EDE-Q. Parallel analysis (PA) and subsequent exploratory factor analysis (EFA) suggested that the adolescent version of the EDE-Q comprises one underlying factor in young adolescent girls (both clinical and general population), centred on Dissatisfaction with Shape and Weight. In boys three factors were found: Weight-related Concerns, Body Discomfort and Restraint.

**Conclusions:**

The EDE-Q appears to measure different underlying aspects of eating disorder psychopathology in young teenagers compared to adults. The dimensions underlying disordered eating in young girls may become increasingly differentiated with time. There appear to be important gender-based differences in the dimensions underlying the EDE-Q in young teenagers. Therapists should be alert to beliefs that girls have about the importance of shape and weight, while in boys it may be more important to be attentive to how they feel about their bodies in relation to weight.

## Background

The Eating Disorder Examination Questionnaire (EDE-Q) [1] is perhaps the single most widely used self-report measure of eating disorder (ED) symptoms. Nevertheless, important questions remain about its validity and applicability in relation to particular groups of individuals, especially adolescents of both genders. The EDE-Q comprises four subscales of postulated underlying psychopathology (Restraint, Eating Concern, Shape Concern, and Weight Concern^1^) and measures the occurrence and frequencies of key ED behaviours. The adult version of the instrument has been the subject of numerous studies investigating its reliability and validity in different populations [1–11]. Test-retest reliability is generally reported as good, ranging from 0.66 to 0.94 for subscales and from 0.51 to 0.92 for the key ED behaviour items [7, 10]. The subscales demonstrate acceptable internal consistency with alphas ranging from .70 to .91 [3, 7, 8]. Studies of convergent validity comparing the EDE-Q with its interview equivalent (the EDE) have generally demonstrated good agreement between measures [1, 4, 11].

Despite extensive psychometric work on the adult version of the EDE-Q and a positive picture of reliability and internal consistency, factor analytic studies are highly equivocal. The postulated four-factor structure thought to characterise the instrument’s subscales has never been replicated. Since the subscales were originally based on theoretical assumptions, rather than being empirically derived, this may not be surprising. Using exploratory factor analysis (EFA) Peterson and co-workers [7] found a three-factor model, where Shape and Weight Concerns merged into one factor, and Restraint and Eating Concern remained fairly distinct. In a study of the general population using EFA, Becker and colleagues [5] found support for a two-factor model where Eating, Shape, and Weight Concern collapsed into one factor, while Restraint remained a stand-alone factor. Darcy and co-workers [12] carried out both EFA and confirmatory factor analysis (CFA) on EDE-Q data from male and female college athletes and a control group. For the male control group a two-factor solution provided the best fit, whereas for the other three groups a three-factor solution fit best. Work by Allen and colleagues [2] with CFA in both clinical and community samples only managed to find support for a one-factor model consisting of eight Shape and Weight items. An EFA on an adult clinical Portuguese sample found three factors, roughly representing Shape/Weight concern, Restraint, and Eating concern [13]. Other studies that were unable to support the four-factor structure in adults, have found support for a single factor or a briefer questionnaire in relation to bariatric surgery candidates [14], Chinese and US students [15, 16], as well as Italian in- and outpatients [17]. This broad spectrum of factor analytic findings is a problem, and shows consistency only in not replicating the purported structure of the instrument. It appears impossible to conclude that the EDE-Q as currently used reflects a consistent and uniform underlying structure of psychopathology.

Another important problem is that there is little systematic knowledge about the EDE-Q in relation to adolescent girls and boys. Since adolescents comprise a significant proportion of ED patients it is important to know how well the instrument performs in relation to younger individuals from both clinical and non-clinical samples. Its factor structure has been investigated using British [18] and Mexican [19] adolescents, with both studies failing to find support for the four-factor model using CFA. However, both of these studies used the adult version of the instrument. There is a distinct version of the EDE-Q for use with younger subjects [20]. It diverges from the adult version, focusing on the past 14 rather than 28 days, and uses simplified language more suitable for younger individuals. Unfortunately, research on the adolescent version of the EDEQ has been extremely limited. Only two studies present normative data [20, 21]. Factor analytic studies of the adolescent version of the EDE-Q are needed in order to investigate its underlying structure and whether it shows similar properties to the adult version. This is important since the hypothetical four-factor structure of the EDE-Q is commonly accepted and used in both clinical assessment and for screening purposes in adolescents. Knowledge is also needed about the adolescent version in relation to both normal girls and boys, and in relation to clinical samples. A psychometrically sound adolescent version of the EDE-Q would aid researchers in the estimation of risk and identification of early onset, and would help clinicians to measure key symptoms and monitor treatment results in individual cases.

Given the limited knowledge of the adolescent version of the EDE-Q the present study aimed to investigate its factor structure in Swedish girls and boys from the general population and girls with a diagnosed eating disorder. Since there is no previous study investigating the factor structure of this particular version of the instrument we first used confirmatory factor analysis (CFA) to test the four-factor model, followed by exploratory factor analysis (EFA) if the four-factor model was not supported.

## Method

### Participants

#### General population sample

Data were collected in Swedish public schools, representative of national demographics. A total of 675 adolescent boys and girls (aged 13–15) could have participated in the study. For unknown reasons 171 of these pupils did not attend school on the day of data collection, and 17 of those who did were excluded due to missing data, resulting in data on *N* = 487 (72% of the possible sample, 239 girls and 248 boys). Age ranged from 12 to 14 years (girls: M = 13.46, SD = .50; boys: M = 13.49, SD = .50).^2^ Exact age was not recorded; instead mean age was estimated based on the grade participants were attending. This is the same sample used in our previous work [21].

#### Clinical sample

The ED sample was extracted from the Stepwise database, a large Internet-based quality assurance and data collection system used across Sweden [22]. The database was set up in 2005 and includes more than 40 clinics across the country. Inclusion criteria are medical- or self-referral to one of the participating treatment units, a DSM-IV ED diagnosis, and intention to treat the patient at the unit. There were 785 patients aged 12–14 registered in Stepwise when data were extracted. Of these, 59 male patients were excluded since they were too few to analyse separately using factor analysis. Another 109 patients were excluded due to missing diagnostic data, and 47 due to erroneous registration, i.e. no DSM-IV ED diagnosis was present. The final sample comprised 570 patients (73% of the initially extracted sample). Age ranged from 12 to 14 years with a mean age of 13.46 (SD = .69). DSM-IV diagnoses were: AN (*N* = 255, 44.7%), BN (*N* = 12, 2.1%), EDNOS (*N* = 301, 52.8%), and BED (*N* = 2, 0.4%).

### Measures

The adolescent version of the EDE-Q [20], based on the adult EDE-Q 4.0, was used. There is a later version of the adult questionnaire (version 6.0) [23], but items comprising the subscales are unchanged. The adolescent version focuses on the past 14 days and respondents rate each of the 36 items on a 7-point rating scale, where higher scores denote more problematic eating behaviours and attitudes. Like the adult version, the instrument generates four subscale scores and a global score, which is the average of the four subscales. Frequencies of key ED behaviours (compensatory behaviours and binge eating) are assessed by respondents specifying how often particular behaviours occurred during the past 14 days. These items are not used to compute subscale scores. Since the instrument was administered in Sweden, a valid translation was required. This was done following the Swedish translation procedure used with the adult version of the EDE-Q [24].

### Procedure

In the general population sample, final term MSc (psychology) university students administered self-report questionnaires in class during school hours. The research assistants followed a manual for administration, and informed participants that participation was voluntary and their responses confidential. Data collection was conducted during a 2-week period. Parents were informed about the project and encouraged to contact the project supervisor if they did not want their child to participate. No parents objected.

In the clinical sample, ED professionals assessed patients using the Stepwise system. Patients were assessed prior to treatment and no later than at their third visit to the treatment unit in question. Patients (and parents) were informed about Stepwise and about research use of data being voluntary. The assessment starts with MINIKid DSM-IV Axis I screening interview [25] followed by the SEDI [22], clinical ratings of level of functioning and ED severity, and finally self-report measures (EDE-Q being first in line, followed by psychological symptom and self-image measures that are not considered here). The entire assessment takes approximately 45 min for this age group.

### Statistical analysis

The four-factor model of the EDE-Q was tested by conducting confirmatory factor analysis (CFA) in each of the three samples. This was done using the Lavaan package version 0.5–13 for R [26], version 3.0.0 for Mac [27]. Indices were the comparative fit index (CFI) and Tucker–Lewis Index (TLI; exact fit = 1.00, close fit = 0.95–0.99, acceptable fit = 0.90–0.95) [28] as well as the root mean square error of approximation (RMSEA; exact fit = 0.00, close fit = 0.01–0.06, acceptable fit = 0.06–0.08) [29]. Exploratory factor analysis (EFA) was performed on the 22 EDE-Q items allocated to subscales using principal axis factoring (PAF) as the method of extraction, and subsequent oblique (promax) rotation. Prior to EFA parallel analysis (PA) [30] was conducted to determine the number of factors to retain for each sample. This is one of the most robust methods for determining the optimal number of factors to retain [31]. PA generates a random set observations and variables equal to the number in the observed dataset. Using principal components analysis, eigenvalues are extracted from the random data, and this is repeated for 1000 iterations. Eigenvalues from the 95th percentile of the random set are then compared to those from the observed data, and those with eigenvalues greater than the random data are retained. EFA was carried out using SPSS Statistics version 24, and PA utilized SPSS syntax developed by O’Connor [32]﻿.

## Results

Descriptive data on age and coefficients of internal consistency (Chronbach’s alpha) for EDE-Q subscale scores in the three samples are presented in Table 1. Not unexpectedly, clinical girls scored higher than boys and girls from the general population on all EDE-Q subscales, and girls from the general population scored higher than boys on all subscales. Internal consistency was good for all subscales, although somewhat lower for boys compared to both samples of girls.

**Table 1:** Age and Chronbach’s alpha for EDE-Q subscales samples of general population girls and boys and clinical girls

	General population girls (*n* = 239)		General population boys (*n* = 248)		Clinical girls (*n* = 570)	
	M (SD)	*alpha*	M (SD)	*alpha*	M (SD)	*alpha*
Age	13.46 (.50)		13.49 (.50)		13.46 (.69)	
EDEQ Global	1.41 (1.36)	*.96*	.59 (.82)	*.93*	3.03 (1.71)	*.96*
Restraint	1.09 (1.40)	*.86*	.47 (.90)	*.77*	2.92 (2.00)	*.87*
Eating concern	.86 (1.10)	*.77*	.37 (.71)	*.75*	2.33 (1.56)	*.77*
Shape concern	2.03 (1.74)	*.93*	.80 (1.11)	*.88*	3.77 (1.92)	*.94*
Weight concern	1.68 (1.63)	*.87*	.70 (1.06)	*.80*	3.12 (1.88)	*.87*

### Confirmatory Factor Analysis (CFA)

CFA for normal boys failed to converge after 10,000 iterations, suggesting that the four-factor model is markedly discrepant from the structure of the data in boys. In the samples of girls (both population-based and clinical) CFA did converge (Table 2). RMSEA > .10 and CFI/TLI < .90 suggested poor fit.

**Table 2:** Results of CFA - goodness of fit for the four-factor model of the EDE-Q for general population and clinical girls

	General population girls	Clinical girls
RMSEA	0.11	0.11
TLI	0.85	0.85
CFI	0.87	0.87

*RMSEA* Root Mean Square Error of Approximation*, TLI* Tucker-Lewis Index, *CFI* Comparative Fit Index

Since CFA failed to provide evidence of good fit for samples of girls and boys based on the original four-factor model of the EDE-Q, exploratory factor analysis (EFA) was conducted to investigate alternative factor structures.

### Exploratory Factor Analysis (EFA)

PA and EFA were conducted separately in the three samples. PA suggested that the optimal number of factors to retain was only one in both samples of girls, and three in general population boys. Kaiser-Myer-Olkin measures of sampling adequacy (KMO) were good: normal girls KMO = .94 (Bartlett’s Test of Sphericity *p* < .001); normal boys KMO = .89 (Bartlett’s Test of Sphericity *p* < .001); and clinical girls KMO = .95 (Bartlett’s Test of Sphericity *p* < .001).

Principal axis factoring (PAF) with oblique promax rotation was conducted in each sample with the number of factors retained limited to the number suggested by the previous PA. In order to optimally delineate the underlying factor structure in each sample a series of steps were undertaken based on the recommendations of Costello and Osborne [33]. First, communalities were examined and items with low values (i.e. < .30) were eliminated from subsequent analyses. This resulted in the elimination of item 9 in all three samples, along with item 31 in the sample of general population boys. Based on recommendations by Costello and Osborne [33] items were also removed from further analyses if loadings were less than .40, or cross-loadings were .32 or above [34]. Since factor structures were stable in the two samples of girls after removal of item 9, and no items met criteria for removal based on weak loadings or cross-loadings no further analyses were conducted in these samples. In the sample of boys, however, PAF with promax rotation was rerun and items 6, 7, 11, 29 and 30 were removed because of cross-loadings > .32, and item 12 was deleted because of factor loading < .40. In a final step in the sample of boys PAF was rerun resulting in the removal of item 5 because of low loadings. At this point the factor structure in the sample of boys was stable. Results of factor analysis reported in Table 3.

**Table 3:**
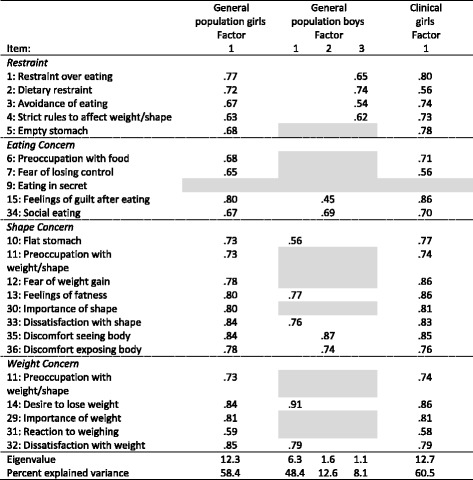
Results of EFA using principal axis factoring, preceded by parallel analysis to determine number of factors, and following promax rotation (pattern matrix) when appropriate and stepwise removal of items (*grey cells*) based on primary factor loadings (< .4) and cross-loadings (≥ .32)

Note that item 11 is used in both the Shape Concern and Weight Concern subscales, and that results for this item are repeated in the table in order to make patterns of factor loadings in relation to subscales more discernable

Results of EFA in both clinical and general population girls were highly unitary, with both samples characterised by one large factor explaining approximately 60% of the variance in each sample, and most individual items loading highly on each factor. Since there was a tendency for items concerning dissatisfaction with shape and weight to have the highest loadings on the two general factors in girls, the factor was labelled Dissatisfaction with Shape and Weight in both samples. The factor structure in boys was considerably more diverse, with a large factor that was somewhat similar to the one found in the samples of girls, but with more of a focus on weight, and was labelled Weight-related Concerns. The second factor centred on items discomfort in relation to their bodies and difficulties in relation to eating; it was labelled Body Discomfort. The third factor in boys comprised items from the Restraint subscale and was accordingly labelled Restraint.

## Discussion

The present study aimed to investigate the validity of the adolescent version of the EDE-Q by examining its factor structure in samples of population-based and clinical Swedish young adolescents. Although there has been considerable factor analytic work on the adult version of EDE-Q and a small number of studies examining it in relation to adolescents, the present study is unique in that it tests the adolescent version of the EDE-Q in large samples of both girls and boys from the general population, and girls with a diagnosed eating disorder. This increases the generalizability of the study, and makes for a better evidence base for conclusions about underlying dimensions of eating pathology in population-based and clinical samples of adolescents.

Results of CFA failed to support the four-factor model of the EDE-Q. These findings are in keeping with a growing body of evidence in relation to the adult version of the EDE-Q that has failed to confirm the four-factor model of the questionnaire [2, 12, 28, 35, 36]. Using PA to determine the optimal number of factors in each sample, our results suggest that in both samples of adolescent girls a single factor centring on Dissatisfaction with Shape and Weight is sufficient to explain well over half the variance in the adolescent version of the EDE-Q. Studies of the adult version of the instrument in university students have tended to find three factors (e.g. Darcy et al) [12]. White and colleagues [18] who studied a notably young sample between 14 and 18 years of age also found a three-factor structure in adolescents. Both these studies utilised PAF, as was done in the present study. However, only the study by Darcy and colleagues utilised PA to determine the number of factors to retain for further analysis. White and colleagues used traditional Kaiser criteria and retained factors with eigenvalues greater than 1. Given that such methods may overestimate or inconsistently identify the number of factors in a data set [33], White and colleagues [18] may have inadvertently retained too many factors. Interestingly, the PA used in the present study closely followed the procedures adopted by Darcy et al. [12], which raises the question of why we found only one factor in our samples of girls.

One possible reason for the discrepancy between our results for girls and those found by Darcy’s [12] group could be the age of the two samples. Mean age in our data was 13.5 years for both samples of girls, while mean age in Darcy’s [12] samples were all around 21 years. It may be that issues relating to shape, weight and eating, although important to young teenage girls, are less differentiated during these earlier years. In adolescence, restrictive eating pathology is most common, with bulimic and other ED symptoms becoming more prevalent in young adulthood [37]; hence both underlying issues and actual ED behaviours are possibly more one-dimensional in younger girls. Our results imply that it may be important to take developmental factors into account when considering problematic aspects of shape, eating and weight in girls. During earlier years these concerns may tend to be global in nature. As these girls grow older the same concerns might tend to differentiate and develop into the underlying structures that have been found in studies of the EDE-Q focusing on three factors. Such a developmental perspective may also have implications for prevention, and suggests that early interventions in girls might usefully target self-esteem in relation to general ideas about shape and weight.

Boys from the general population scored by far the lowest on the all EDE-Q subscales and had the most diverse factor structure, which suggests some considerable gender differences from early teenage years. When boys develop issues related to disordered eating at an early age these may tend to centre on weight concern, discomfort in relation to their bodies and restrained eating. These differences imply a complexity of weight-related concerns in relation to gender. For normal boys, eating disorder related concerns may be more narrow and specific, focusing on dissatisfaction with perceived over- or even underweight (as in Factor 1) and eating in a more restrained manner (Factor 2), whereas for girls, body, weight and eating may relate more generally to who they experience themselves to be as individuals, i.e. more global self-worth. In other research, we have shown that the association between self-image and eating disorder symptoms is several times stronger in girls as compared to boys [21].

When assessing weight-related issues, it may be valuable to keep these gender differences in mind. Clinically, it may be important for therapists to be alert to young teenage girls having undifferentiated beliefs about the central importance of shape and weight for self-esteem, while in boys these concerns may be more domain-specific and less impactful. When considering gender differences in young adolescents it is important to note that one third of the items had to be deleted in boys in order to attain a stable factor structure. In both samples of girls only one item (eating in secret) was deleted. This raises the possibility that the EDE-Q is of limited utility in relation to young teenage boys.

It is interesting that the item concerning secretive eating was the only one to be excluded from the factor analysis of all three samples, with markedly low communalities. Although this item appears to have little relevance to the underlying dimensions of disordered eating at a young age, it raises the possibility that secretive eating might be useful as an early warning sign of a potential eating disorder, given that the item is included in factor analyses of older and clinical samples. This could be tested in future longitudinal research. Future studies could also examine factors that may mediate or moderate the early form of shape and weight concern found in adolescents. Given the universal relevance of the Dissatisfaction with Shape and Weight factor for both samples of girls, it may be important to pay it closer attention in community-based research endeavouring to chart risk factors longitudinally. For example, future studies could examine factors that may mediate or moderate shape and weight concern over time in different groups of adolescents.

Although the present study has the advantage of testing the adolescent version of the EDE-Q in relatively large samples of girls and boys from the general population, along with cases of diagnosed eating disorders in girls, it also has weaknesses. The factor structures we found through EFA may be sample-specific, hence not generalizable to other samples. Nevertheless, our study is a starting point for evaluating the factor structure of the adolescent version of the EDE-Q, and we encourage other researchers to conduct EFA in other samples of adolescents. Another weakness of the present study concerns the sample of clinical boys, which was too small to include in the present analyses. It therefore remains to be seen if clinical boys show similar patterns to other groups (and in particular boys from the general population). The present study could also have been affected by the relatively high degree of non-attendance at school on the day the EDE-Q was administered. It has been found that body image dissatisfaction may lead to absence from school [38], which could mean that some pupils with higher levels of eating psychopathology were underrepresented. Our work, nevertheless, widens the evidence base, arguing against using the EDE-Q as a four-subscale measure, provides indicators for future research, and raises some important clinical questions about gender and age differences in underlying ED psychopathology.

## Conclusion

The factor structure of the adolescent version of EDE-Q in young teenage girls centres on a single underlying dimension of dissatisfaction with shape and weight. This appears to be the case in both clinical and general population samples. Over time, this general factor may become increasingly differentiated, giving way to the more complex patterns found in older girls and women. There appear to be important gender-based differences in the dimensions underlying the EDE-Q in young adolescents. Therapists should be alert to beliefs that girls have about the importance of shape and weight for their self-image at an early age. Weight-related concerns in young teenage boys may not have the same overall impact on self-image, but therapists should nevertheless be prepared to address these directly in treatment if and when they arise.

## Abbreviations

AN: Anorexia Nervosa
CFA: Confirmatory factor analysis
ED: Eating disorder
EDE-Q: Eating Disorders Examination Questionnaire
EFA: Exploratory factor analysis

## Footnotes

1 Although in the study routinely cited as the source of the EDE-Q (i.e. Fairburn and Beglin 1994), only three subscales are mentioned; Eating concern is not included. The four subscales that have since become the norm stem from the 12th edition of the Eating Disorders Examination interview (Fairburn and Cooper,1993). According to Fairburn and Beglin (1994), the EDE-Q was based on the 11th EDE version and comprised three of its five subscales, besides the behavioural items. The four-subscale variant appears to be a later adaptation.

2 Grade 7 (13–14 years of age); Males = 49.4%, Females = 50.6%, Grade 8 (14–15 years of age); Males = 52.6%, Females = 47.4%.

## References

[1] Fairburn CG, Beglin SJ (1994). Assessment of eating disorders: interview or self-report questionnaire?. Int J Eat Disorder.

[2] Allen KL, Byrne SM, Lampard A, Watson H, Fursland A (2011). Confirmatory factor analysis of the eating disorder examination questionnaire (EDE-Q). Eat Behav.

[3] Bardone-Cone AM, Agras WS (2007). Psychometric properties of eating disorder instruments in black and white young women: internal consistency, temporal stability, and validity. Psychol Assess.

[4] Black CMD, Wilson GT (1996). Assessment of eating disorders: interview versus questionnaire. Int J Eat Disorder.

[5] Becker AE, Thomas JJ, Bainivualiku A, Richards L, Navara K, Roberts AL, Gilman SE, Striegel-Moore RH (2010). Validity and reliability of a Fijian translation and adaptation of the Eating Disorder Examination Questionnaire. Int J Eat Disorder.

[6] Brundin Pettersson C, Zandian M, Clinton D (2016). Eating disorder symptoms pre- and postpartum. Arch Womens Ment Health.

[7] Luce KH, Crowther JH (1999). The reliability of the Eating Disorder Examination-Self-Report Questionnaire Version (EDE-Q). Int J Eat Disorder.

[8] Mond JM, Hay PJ, Rodgers B, Owen C, Beumont PJV (2004). Validity of the eating disorder examination questionnaire (EDE-Q) in screening for eating disorders in community samples. Behav Res Ther.

[9] Peterson CB, Crosby RD, Wonderlich SA, Joiner TE, Crow SJ, Mitchell JC, Bardone-Cone AM, Klein M, Le Grange D (2007). Psychometric properties of the eating disorder examination-questionnaire: factor structure and internal consistency. Int J Eat Disorder.

[10] Reas DL, Grilo CM, Masheb RM (2006). Reliability of the eating disorder examination-questionnaire in patients with binge eating disorder. Behav Res Ther.

[11] Wilfley DE, Schwartz MB, Spurrel EB, Fairburn CG (1997). Assessing the specific psychopathology of binge eating patients: Interview or self-report?. Int J Eat Disorder.

[12] Darcy AM, Hardy KK, Crosby RD, Lock J, Peebles R (2013). Factor structure of the eating disorder examination questionnaire (EDE-Q) in male and female college athletes. Body Image.

[13] Machado PP, Martins C, Vaz AR, Conceicao E, Bastos AP, Goncalves S (2014). Eating disorder examination questionnaire: psychometric properties and norms for the portugese population. Eur Eat Disord Rev.

[14] Parker K, Mitchell S, O’Brien P, Brennan L (2015). Psychometric evaluation of disordered eating measures in bariatric surgery patients. Eat Behav.

[15] Chan CW, Leung SF (2015). Validation of the EDEQ: an online version. J Hum Nutr Diet.

[16] Grilo CM, Reas DL, Hopwood CJ, Crosby RD (2015). Factor structure and construct validity of the EDEQ in college students: further support for a modified brief version. Int J Eat Disorder.

[17] Calugi S, Milanese C, Sartirana M, El Ghoch M, Sartori F, Geccherle E, Coppini A, Frnachini C, Dalle Grave R. The eating disorder examination questionnaire: reliability and validity of the Italian version. Eat Weight Disord. 2016; doi:10.1007/s40519-016-0276-6. 10.1007/s40519-016-0276-6 27039107

[18] White HJ, Haycraft E, Goodwin H, Meyer C (2014). Eating Disorder Examination Questionnaire: Factor structure for adolescent girls and boys. Int J Eat Disorder.

[19] Penelo E, Negrete A, Portell M, Raich RM (2013). Psychometric properties of the Eating Disorder Examination Questionnaire (EDE-Q) and norms for rural and urban adolescent males and females in Mexico. PLoS One.

[20] Carter JC, Stewart DA, Fairburn CG (2001). Eating disorder examination questionnaire: norms for young adolescent girls. Behav Res Ther.

[21] Forsén Mantilla E, Birgegård A (2015). Eating disorder examination questionnaire: Norms and clinical reference data from adolescent boys and girls in Sweden. Psychiatry Res.

[22] Birgegård A, Björck C, Clinton D (2010). Quality assurance of specialized treatment of eating disorders using large-scale internet-based collection systems: methods, results and lessons learned from designing the Stepwise database. Eur Eat Disord Rev.

[23] Fairburn CG, Beglin SJ (2008). Eating Disorder Examination Questionnaire (6.0). In Fairburn CG, editor. Cognitive behaviour therapy for eating disorders.

[24] Welch E, Birgegård A, Parling T, Ghaderi A (2011). Eating Disorder Examination Questionnaire and clinical impairment assessment questionnaire: general population and clinical norms for young adult women in Sweden. Behav Res Ther.

[25] Sheehan DV, Lecrubier Y, Sheehan KH, Amorim P, Janavs J, Weiller E (1998). The MINI-international Neuropsychiatric Interview (MINI): the development and validation of a structured diagnostic psychiatric interview for DSM-IV and ICD-10. J Clin Psychiatry.

[26] Rosseel Y (2012). LAVAAN: An R Package for Structural Equation Modeling. J Stat Softw.

[27] R Core Team. R: A language and environment for statistical computing. R Foundation for Statistical Computing, Vienna, Austria; 2013.

[28] Bentler PB, Bonett DG (1980). Significance tests and goodness-of-fit in the analyses of covariance structures. Psychol Bull.

[29] Browne MW, Cudeck R (1993). Alternative ways of assessing model fit. Sociol Methods Res.

[30] Horn JL (1965). A rationale and test for the number of factors in factor analysis. Psychometrika.

[31] Velicer WF, Eaton CA, Fava JL. Construct explication through factor or component analysis: A review and evaluation of alternative procedures for determining the number of factors or components. In Goffin RD & Helmes E, editors. Problems and solutions in human assessment; 2010.

[32] O’Connor BP (2000). SPSS and SAS programs for determining the number of components using parallel analysis and Velicer’s MAP test. Behav Res Methods.

[33] Costello AB, Osborne JW. Best practices in exploratory factor anal- ysis: Four recommendations for getting the most from your analysis. Practical Assessment. Res Eval. 2005;10:1–9.

[34] Tabachnick B, Fidell L (2001). Using multivariate statistics.

[35] Byrne SM, Allen KL, Lampard AM, Dove ER, Fursland A (2010). The factor structure of the Eating Disorder Examination in clinical and community samples. Int J Eat Disorder.

[36] Wade TD, Byrne S, Bryant-Waugh R (2008). The Eating Disorder Examination: norms and construct validity with young and middle adolescent girls. Int J Eat Disorder.

[37] Wijbrand Hoek H, van Hoeken D (2003). Review of the prevalence and incidence of eating disorders. Int J Eat Disorder.

[38] Yanover T, Thompson JK (2008). Eating problems, body image disturbances, and academic achievement: Preliminary evaluation of the eating and body image disturbances academic interference scale. Int J Eat Disorder.

